# Eliminating Plastic Anisotropy of TiB-Reinforced Titanium Matrix Composite via Cross Rolling

**DOI:** 10.3390/ma18050990

**Published:** 2025-02-24

**Authors:** Kuangzhe Xia, Kening Chen, Junyang He, Xiaoyong Zhang, Kechao Zhou

**Affiliations:** State Key Laboratory of Powder Metallurgy, Central South University, Changsha 410083, China; 223312110@csu.edu.cn (K.X.); 233303040@csu.edu.cn (K.C.); junyanghe@csu.edu.cn (J.H.); zhoukechao@mail.csu.edu.cn (K.Z.)

**Keywords:** titanium matrix composites, TiB whisker, plastic anisotropy, orientation

## Abstract

Anisotropy is one of the concerns of titanium matrix composites (TMCs) due to its impact on subsequent processing and safe serving. In this work, we demonstrate that simply by using cross-rolling (CR) rather than unidirectional rolling (UDR), significant plastic anisotropy can be removed and an excellent strength–ductility combination can be achieved in a new TiB-reinforced TMC (TiB-TMC). We attribute the occurrence of plastic anisotropy after UDR to be the strong rolling-induced orientation distribution of TiB whiskers parallel to the rolling direction (RD), while the elimination of anisotropy is therefore confirmed to be caused by the loss of such an orientation relationship. The underlying mechanism is revealed as the lower stress concentration along the lateral surface of the randomly distributed whiskers and the longer paths when cracks propagate in between each whisker, in the CR-processed TiB-TMC.

## 1. Introduction

TMCs have great potential in the aerospace, weapons, and automobile fields because of their excellent mechanical properties [1,2,3]. TiB, known as a classical titanium compound with an orthorhombic crystal [4,5], has a coefficient of thermal expansion similar to the matrix and is thermally super-stable, making it one of the most suitable reinforcements for TMCs [6,7,8]. TiB can be precipitated and distributed in situ [9,10] in the titanium alloy matrix. TiB often presents in the form of whiskers (which grow along the [010] direction) and forms at the original β grain boundary due to the extremely low solution limit of boron in the titanium lattice [11]. Hot rolling is always essential for manufacturing TMCs, considering benefits including eliminating casting defects and refining the grain [12]. However, as-rolled plates often display anisotropy, which not only influences the subsequent processing routes [13] but also causes serious trouble for safe service [12,14] where multiaxial loading is inevitable. Therefore, anisotropy has been considered an important research topic in the design of TMCs.

As for TiB-TMC, the primary factor determining the strength anisotropy is the as-rolled matrix texture [15]. Two basic types of texture [16] frequently observed and reported in titanium alloys are the T-type texture (crystal c-axis orientation towards transverse direction, TD) and the B-type texture (crystal c-axis orientation towards normal direction, ND). Li et al. [17] found that for the T-type texture, TD loading has lower Schmidt factors (SFs) [18] than RD loading, making it difficult to activate the slip system and hence results in strength anisotropy. On the contrary, the B-type texture shows similar SFs in both rolling directions, resulting in comparable deformation capabilities and weakened anisotropy.

Notice that TiB itself must be seen as anisotropic considering the whisker shape [4] in TiB-TMC. What makes it worse is that these TiB whiskers are brittle in nature and easily fracture upon loading [19], acting as nucleation sites for microcracking in the surrounding alloy matrix and therefore deteriorating the composite plasticity [20,21]. In this way, the existence of TiB reinforcements may also contribute to the overall anisotropy of the as-rolled plates. Zhang et al. [22] prepared TMCs with a uniformly oriented distribution of TiB whiskers by extrusion molding, and the material showed good plasticity in the extrusion direction yet presented premature failure when there was extruded transverse loading. However, the underlying mechanism of the anisotropy caused by TiB whiskers and how to quantify and further avoid the detrimental effects originating from such anisotropy still remain open questions.

To address the above concerns, in this work, we designed a new TiB-TMC and prepared as-rolled plates with different TiB distribution characteristics by UDR and CR. The tensile properties of the TiB-TMC were carefully investigated along the RD and TD of the UDR plates and along both rolling direction 1 (RD1) and rolling direction 2 (RD2) of the CR plates. Our results indicated that UDR induced significant plastic anisotropy while CR perfectly removed it. By digging into the microstructural responses under the different conditions mentioned above, a thorough understanding of the effect of whisker arrangement on plastic anisotropy was successfully achieved, with particular emphasis on the stress concentration along the lateral surface of the TiB whisker that initiated the matrix cracks and the interspacing between each whisker which, in general, determined the crack length.

## 2. Experiment

**Figure 1:**
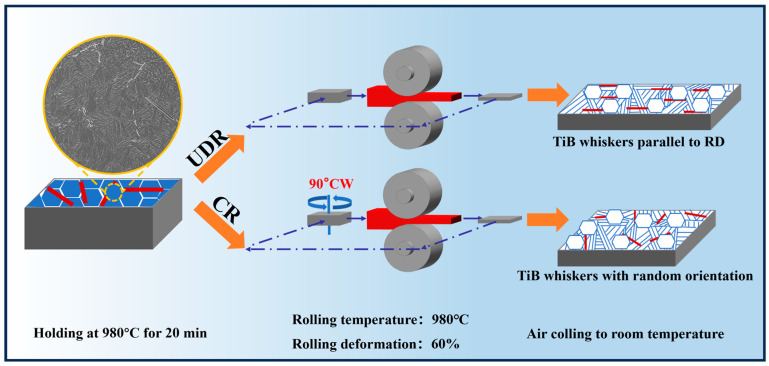
A schematic of the rolling processes.

A new TiB-TMC (Ti-8Al-1Cr-1V-0.5Fe-0.1Si-0.1B), namely LD-Ti423B, was prepared by vacuum self-consumption arc melting. In the following, the alloy ingot was open-die forged above the (α + β)/β transformation temperature (1015 °C according to the metallographic method), presenting a lamellar microstructure before rolling. The forged ingot was then cut into plates using an electric discharge machining (EDM) cutting machine (Suzhou Renguang Numerical Control Equipment Co., Suzhou, China). A schematic diagram of the hot rolling process is presented in Figure 1. Hot rolling was subsequently conducted at a temperature of 980 °C, and six passes were carried out for a 60% reduction. The packs were rolled by 10% in each pass and reheated at 980 °C for 5 min between two passes. After, the final pass plates were air-cooled to room temperature. A dog bone tensile specimen with a gauge size of 78 mm × 10 mm × 1.5 mm was excised from the heat-treated samples. Room temperature tensile tests were carried out using an Instron 3369 mechanical testing machine (Instron, Norwood, MA, USA), with a stretching speed of 2 mm/min. The displacement of the specimen was recorded using a YYJ-4/10-L extensometer (Suzhou Shenghui Precision Instrument Technology Co., Ltd., Suzhou, China) and at least 5 replicate experiments for each alloy were performed to ensure the data’s reliability. Subsequently, these pieces were subjected to mechanical grinding with 240 #, 400 #, and 600 # metallographic sandpaper, followed by electrochemical polishing in Kroll’s solution (5% perchloric acid, 35% butanol, and 60% methanol) for 20 s. The microstructures before and after tensile testing were examined using scanning electron microscopy (TESCAN, Brno, Czech Republic) and electron backscatter diffraction (Hitachi Limited, Tokyo, Japan). Thin films for transmission electron microscopy (TEM) were prepared by the means of the electrolytic polishing (Denmar Struers A/S, Copenhagen, Denmark) of Kroll’s solution at −23 °C using a double-jet electrolytic apparatus, and TEM observations were conducted under F20X field emission transmission electron microscopy (TESCAN, Brno, Czech Republic). The obtained imaging data were processed and analyzed using analytical software including Image-J 1.54d and TSL OIM analysis 8.

## 3. Results and Discussion

### 3.1. Microstructures’ Characteristics

**Figure 2:**
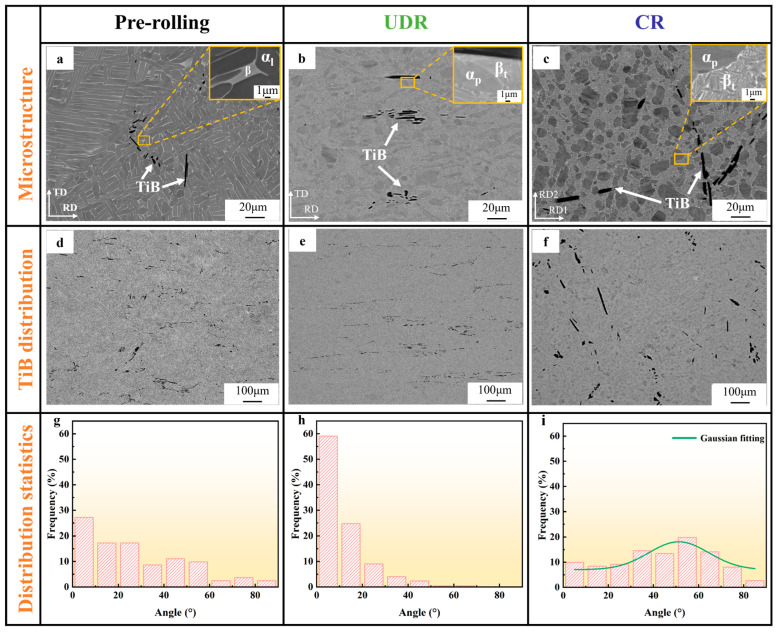
Overall microstructural details of target TiB-TMCs. SEM images showing (**a**–**c**) matrix phase distributions and (**d**–**f**) TiB whisker distributions. (**g**–**i**) Statistics of angle between TiB and RD (RD1), (**a**,**d**,**g**) pre-rolling, (**b**,**e**,**h**) UDR, and (**c**,**f**,**i**) CR.

Figure 2 illustrates the microstructures of the TiB-TMC alloy in different rolling states. Besides the TiB whiskers, in the pre-rolling state (Figure 2a), the alloy matrix primarily consisted of the coarse lamellar α phase (α_l_) and the inter-lamellar β phase. After rolling at a temperature of 980 °C in both the UDR and CR states (Figure 2b,c), the matrix similarly transferred into a composition of the primary α phase (α_p_) and transformed β phase (β_t_), where β_t_ was composed of much finer α_l_ and inter-lamellar β phases (with an average lamellar width refined down to 0.3 μm). The major differences lay in that in the UDR states, both the α_p_ and β_t_ domains appeared elongated along the RD, while those in the CR states stayed near-spherical, apparently as a consequence of the changed rolling directions during CR [23].

When turning to the TiB whiskers, in Figure 2d–f, we, in general, acknowledge the evolution of its orientation distribution from random in the pre-rolling state (actually, these whiskers formed along the primary β grain boundaries during solidification [10,11]) to mostly parallel to the RD in the UDR state or to near-random in the CR state. To quantify such evolution, we measured and listed the distribution of angles between the whiskers and the RD (RD1), as shown in the charts in Figure 2g–i, respectively. It is clear that in the UDR state, over 85% of TiB whiskers presented an angle to the RD of less than 20 degrees. While in the CR state, although the whiskers were indeed more randomly distributed in orientation, a weak Gauss peak near 50 degrees was detected. This was reasonable considering that the CR may have forced the whiskers to weakly align in between the two RDs.

**Figure 3:**
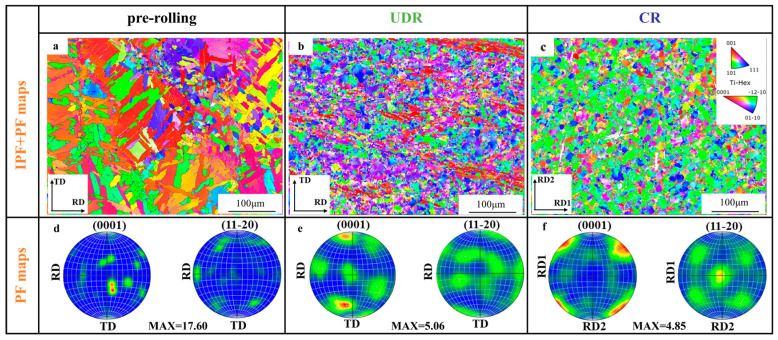
(**a**–**c**) IPF maps and (**d**–**f**) pole figures of target TiB-TMCs, (**a**,**d**) pre-rolling, (**b**,**e**) UDR, and (**c**,**f**) CR.

Figure 3 presents the texture characteristics of the three states via EBSD. Figure 3a–c show the inverse pole figures, while Figure 3d–f display the corresponding pole figures for the (0001) and (11-20) crystal axes. In the pre-rolling state, the alloy exhibited a strong B-type texture where the crystal c-axis aligned along the ND, with a maximum intensity of 17.6 multiples of the uniform density (Mud). In the UDR state, the c-axis orientation shifted from the ND to the TD, making a T-type texture, and the maximum texture intensity decreased to 5.06 Mud. When the rolling mode changed to CR, the maximum texture intensity further decreased to 4.85 Mud, and the crystal c-axis deflected approximately 45 degrees from the TD to the RD. It is seen that both the UDR and CR samples exhibited only weak texture, and the reason could have been due to the existence of the distributed TiB whiskers that restricted the plastic flow during rolling [24], inhibiting the orientation-shifting process. And, other factors affecting the texture intensity include TiB whiskers acting as nucleation sites [25] for recrystallized grains and weakening the texture intensity.

### 3.2. Mechanical Properties and Fracture Morphology

**Figure 4:**
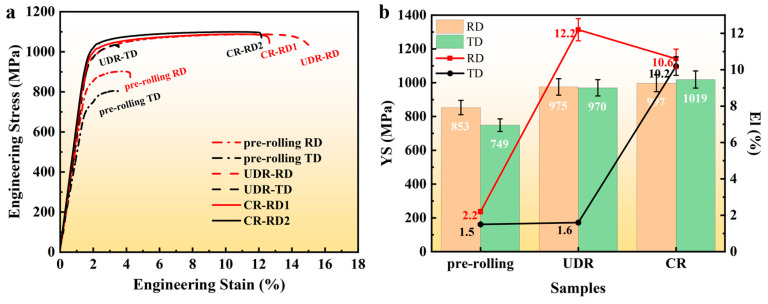
(**a**) Room temperature tensile engineering stress–strain curves and (**b**) variation trends of extracted tensile property indexes including yield stress and elongation.

Figure 4a gives the engineering stress–strain curves, with key mechanical property indexes extracted from these curves and then plotted in Figure 4b. In the pre-rolling state, the yield strength and the elongation measured from the two directions (corresponding to the RD and TD settled in the following rolling) were 853 MPa, 2.2%, and 749 MPa, 1.5%, respectively. The slight strength differences in the two directions in the pre-rolling state was likely due to the strong B-type texture, since the texture significantly influences the initiation of dislocation slip by altering the Schmid factor (SF), thereby directly affecting yield strength through the activation of slip systems [26]. In the UDR state, the yield strength was extremely close for the UDR-RD (975 MPa) and UDR-TD (970 MPa) cases, benefiting from the weakened texture, as revealed in Figure 3. Compared to the pre-rolling state, a large increase in strength was achieved, with Hall–Petch and work-hardening mechanisms playing a dominant strengthening role [27,28]. However, significant plastic anisotropy appeared, with an elongation of 12.2% for the UDR-RD and only 1.5% for the UDR-TD. In contrast, anisotropy, whether in strength or in ductility, totally vanished in the CR sample, with yield strengths of 997 MPa and 1019 MPa and elongations of 10.6% and 10.2%, for CR-RD1 and CR-RD2, respectively.

**Figure 5:**
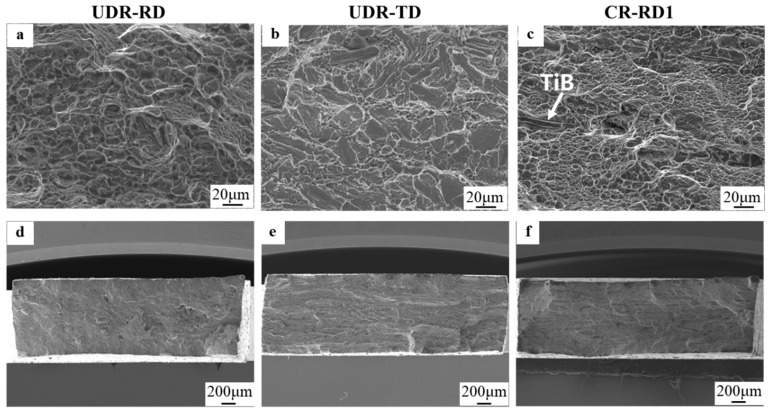
Fracture surfaces of TiB-TMC with different loading directions: (**a**,**d**) UDR-RD, (**b**,**e**) UDR-TD, and (**c**,**f**) CR-RD1.

Figure 5 shows the fracture surfaces of the samples with different loading directions for the two rolling modes, respectively. Since the CR-RD1 and CR-RD2 samples had the same TiB whisker distribution characteristics, only the CR-RD1 sample is presented here. A large number of deep dimples are observed in the UDR-RD and CR-RD1 samples in Figure 5a,c, which indicates that the fracture process of the two samples was ductile fracture [29], in good agreement with the tensile plasticity performances of the two samples. The difference mainly expresses as that, for the UDR-RD, the vast majority of TiB whiskers in the samples were parallel to the tensile direction; therefore, their fracture surfaces were small and difficult to distinguish [30,31]. Meanwhile, for CR-RD1, the randomly tilted TiB whiskers could be found here and there, embedded on the fracture surface. On the contrary, cleavage facets dominated the fracture morphology of the UDR-TD sample (Figure 5b) which indicates that the UDR-TD sample underwent brittle fracture that showed poor plasticity. Notice that, this time, the exposed TiB whiskers on the fracture surfaces were again indistinguishable from the cleavage facets. The exposure of TiB whiskers on the fracture surface indicates the existence of a relationship between crack propagation paths and TiB whiskers during the tensile process of TiB-TMC [32,33,34], which will be further discussed in the following sections.

**Figure 6:**
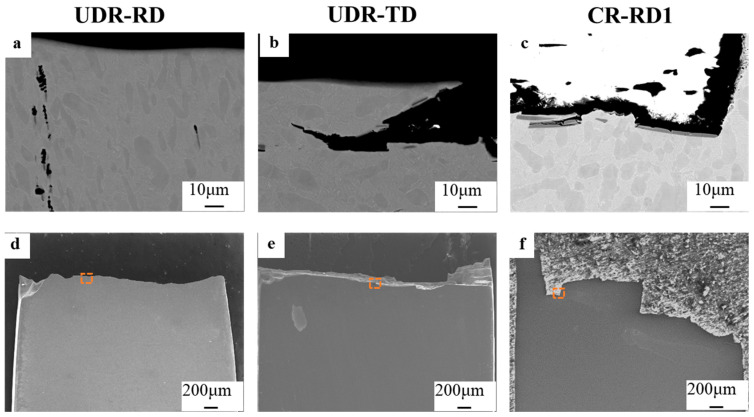
Fracture lateral surfaces of TiB-TMC with different loading directions: (**a**,**d**) UDR-RD, (**b**,**e**) UDR-TD, and (**c**,**f**) CR-RD1.

Figure 6 shows the fracture lateral surfaces of the above three samples under the two rolling modes. Figure 6a–c are magnifications of the squares in Figure 6d–f. TiB whiskers were observed on the tensile fracture surfaces for all these samples (Figure 6a–c), in agreement with the results in Figure 5. This further led to the very tortuous crack path of the CR-RD1 sample, as shown in Figure 6f. When the TiB whiskers within the sample were randomly oriented, the large difference in the orientation of neighboring TiB whiskers more likely led to crack deflection and the formation of zigzag paths.

**Figure 7:**
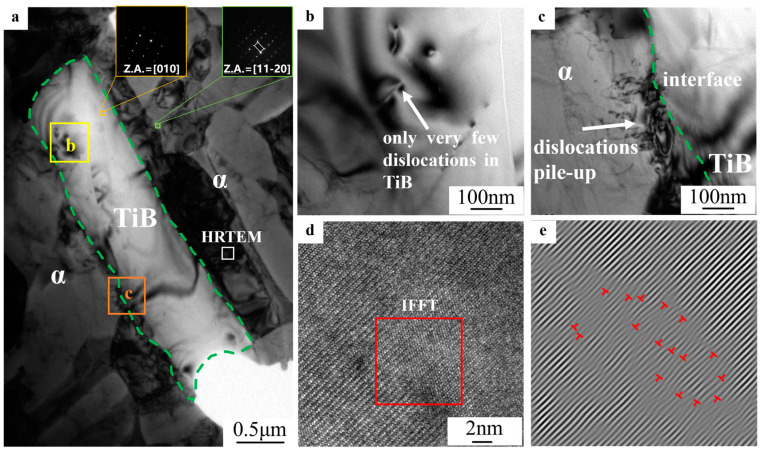
TEM characterization near the tensile fracture region of the UDR-RD sample after tensile tests: (**a**) the sampling position, (**b**) dislocations in the TiB whiskers, (**c**) the dislocations’ pile-up at the surface of the TiB whiskers, (**d**) the HRTEM observation, and (**e**) IFFT images of the matrix.

The fracture analysis indicates that the TiB whiskers had a significant influence on the fracture process of the TiB-TMCs. To figure out the underlying mechanism of the TiB whiskers operating in the alloy, a TEM test was performed near the tensile fracture region of the UDR-RD sample after tensile tests, as shown in Figure 7. Figure 7b shows that there were only a very small number of dislocations within the TiB whiskers. On the contrary, a large number of dislocations were piled up at the matrix/TiB interface (Figure 7c). Figure 7d,e show the HRTEM observation and IFFT images of the matrix, respectively, and a large number of dislocations were present in the matrix. The above results indicate that dislocations hardly transferred between the TiB and matrix and piled up at the matrix/TiB interface, resulting in TiB whiskers firstly fracturing as a consequence of stress concentration along their boundaries.

### 3.3. Effect of TiB Whisker Orientation on Plastic Anisotropy

Considering the similarly weakened overall matrix texture characteristics between the UDR and CR states, and the limited textural contribution to the ductility as proposed in ref. [25], we can briefly attribute the disappearance of anisotropy in the CR state to the changed orientation distribution of the TiB whiskers embedded in the alloy matrix.

**Figure 8:**
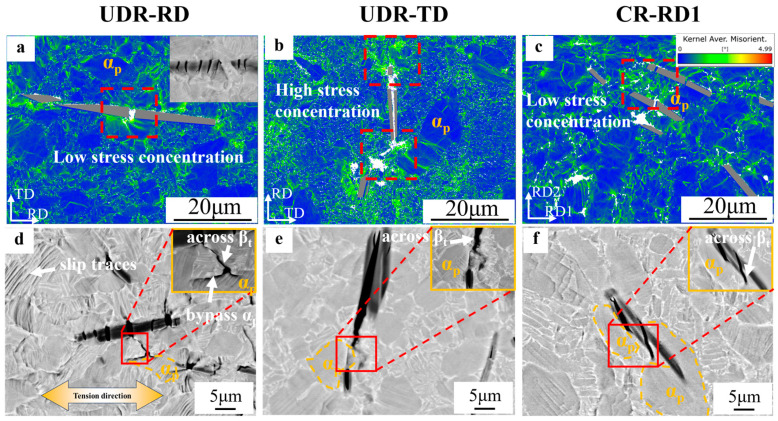
Matrix crack formation and development in the designed TiB-TMC. (**a**–**c**) KAM maps illustrating crack initiation from the vicinity of the TiB whiskers. (**d**–**f**) SEM images showing how cracks propagated inside the matrix once they nucleated in the (**a**,**d**) UDR-RD, (**b**,**e**) UDR-TD, and (**c**,**f**) CR-RD1.

To reveal how TiB distribution affects plastic anisotropy, crack initiation and propagation during tension were carefully investigated, and the quasi-static observation results for both the UDR and CR samples are plotted in Figure 8. We observe that due to the fragile nature [19] of the TiB intermetallic compounds, the TiB whiskers firstly fractured as a consequence of stress concentration [35] along their boundaries (Stage I) and then generated microcracks in the nearby matrix adjacent to the fractured position (Stage II). By further crack propagation and extension, the matrix cracks emitted from different TiB whiskers connected together (Stage III), leading to the final overall fracture.

Figure 8a–c show that in Stage I, in the UDR-TD sample, the TiB whiskers existed perpendicular to the tensile direction, resulting in internal fracture parallel to the long-axis direction. As a consequence, huge stress concentrations developed in the matrix close to the ends of the whiskers, which certainly eased the initiation of the matrix microcracks [36]. In contrast, in the UDR-RD sample, TiB whiskers underwent internal fracture perpendicular to the whiskers’ long-axis direction. This type of perpendicular fracture featured in short cruets and multiple times for each whisker [37] (see the inset in Figure 8a), directly resulting in a much longer stress concentration layer in the vicinity of the entire long-axis-direction boundary of the TiB whisker. This means that the localized stress level around the TiB whisker could be averaged and thus, to a certain degree, reduced, as compared to the case in the UDR-TD sample. In this way, we can propose that the driving force for the nucleation of those matrix cracks in the UDR-RD sample was lower than that in the UDR-TD sample. For the CR sample, on the other hand, since the TiB whiskers were not significantly oriented, the Stage I progress of the CR-RD1 and CR-RD2 samples was essentially the same. Actually, based on our observation, as shown in Figure 8c, the case was much more similar to the UDR-RD sample where multiple fractures in the TiB whiskers together with the averaged stress level around them were probed.

We see in Figure 8d–f that in Stage II, the matrix cracks were prone to propagating across β_t_. This was probably due to the large size of the α_p_ grain being able to induce the long effective slip length and abundant dislocation multiplication so that the strong localized stress concentration [38,39] could be restrained to benefit obstructing crack propagation. In contrast, α_l_, with a small thickness, could hinder dislocation motion, which could benefit crack propagation [40,41].

**Figure 9:**
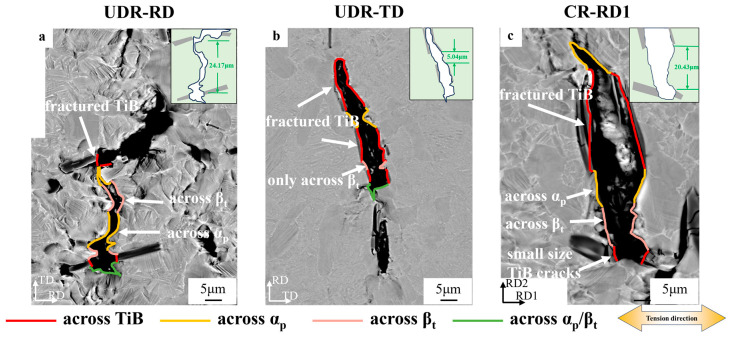
Matrix crack formation and development in the designed TiB-TMCs. (**a**–**c**) SEM images showing how these cracks connected and coalesced when the sample was near fracture: (**a**) UDR-RD, (**b**) UDR-TD, and (**c**) CR-RD1.

Figure 9a–c show that in Stage III, in all investigated alloys, the matrix cracks generated from neighboring TiB whiskers started to meet and coalesce together. The difference between these alloys basically lay in the total length of the cracking path. The reason could be twofold: First is the inter-whisker spacing. Obviously, the lower the value was, the shorter the path to connect two matrix cracks was. We measured the average inter-whisker spacing from the SEM images and we found that the UDR-TD sample had the lowest spacing while the UDR-RD sample had the highest (5.04 μm vs. 24.17 μm). Second is the complexity of the crack path. We happened to notice that if the crack path was shorter, it may have been able to be restricted inside the β_t_ phase with the lowest crack resistance. If the crack further lengthened, it would likely progress and inevitably cross over the α_p_ phase, thereby being obstructed. Taking these two together, we can propose that, due to the higher inter-whisker spacing, the crack propagation in the UDR-RD sample was more difficult as compared to that in the UDR-TD sample. With this wisdom, we noticed that the CR-RD1 and CR-RD2 samples showed nearly the same long paths when cracks lengthened in between TiB whiskers (20.43 μm), making the lengthening resistance of their matrix cracks similar to that in the UDR-RD sample.

**Figure 10:**
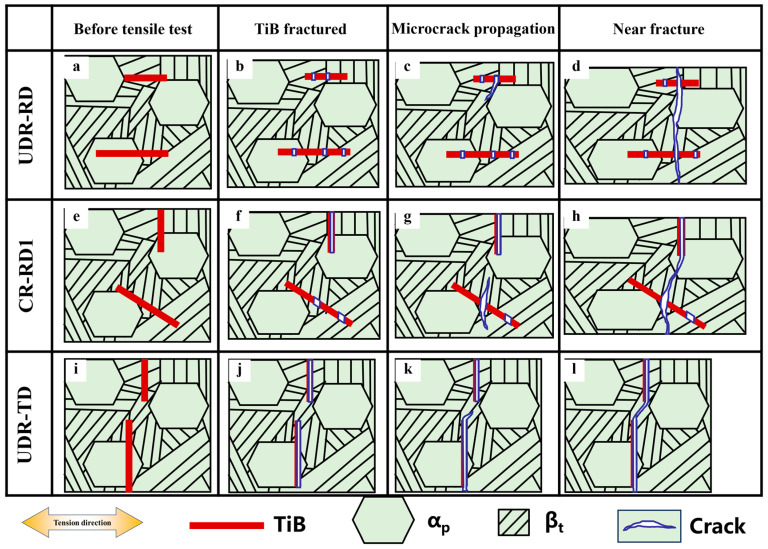
Schematic diagram of crack initiation and propagation via different tensile directions of rolled TiB-TMC during tensile process: (**a**–**d**) UDR-RD, (**e**–**h**) UDR-TD, and (**i**–**l**) CR-RD1.

Figure 10 schematically shows the typical crack initiation and propagation manner during the tensile process for the investigated TiB-TMCs with different TiB distribution characteristics. During tensile deformation, TiB whiskers fractured at the very beginning of the tensile loading, far ahead of those in the matrix due to the inherent brittleness of TiB [17]. The length of the microcracks inside the TiB whiskers was positively correlated with the angle between the TiB whiskers and the direction of tension. As the tensile process proceeded, microcracks in the matrix propagated in β_t_ because the stress concentration in β_t_ was higher than that in α_p_, which facilitated crack initiation and propagation in the matrix. When the tensile process proceeded to near fracture, matrix cracks emitted from different TiB whiskers connected together. For the UDR-RD, CR-RD1, and UDR-TD samples, the average spacing between neighboring TiB whiskers varied due to differences in TiB whisker orientation characteristics. If the crack path was shorter, they may have been able to be restricted inside β_t_ with the lowest crack resistance. And, if the crack further lengthened, it would likely progress and inevitably cross over to α_p_, thereby being obstructed.

Based on the above discussions, we see that considering both the difficulty of matrix crack initiation and the resistance of matrix crack extension, the UDR-RD sample showed higher plastic deformability as compared to the UDR-TD sample. And, due to the same reasons, the plasticity of the CR-RD1 and CR-RD2 samples were consistent, at the same level as that in the UDR-RD sample. In other words, the plastic anisotropy shown in the UDR TiB-TMC alloy disappeared when changing to CR processing, where, excitingly, no strength was sacrificed.

### 3.4. Effects of Texture Characteristics on Strength Anisotropy

The strength anisotropy of the TiB-TMC was weak for both rolling methods. According to the strong correlation between strength anisotropy and matrix texture as reported in the literature [15], texture significantly affects the initiated dislocation slip mode by changing the SFs, resulting in the yield strength being directly related to the activation of deformation mechanisms. SFs are greatly affected by the angular relationship between the loading direction and texture [25]. We attribute weaker strength anisotropy to a weaker matrix texture.

**Figure 11:**
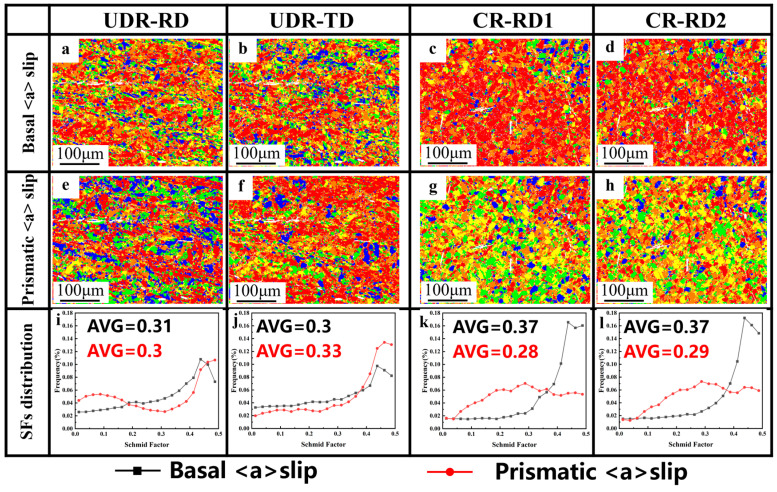
(**a**–**h**) Schmid factor maps and (**i**–**l**) SF distribution: (**a**,**e**,**i**) UDR-RD, (**b**,**f**,**j**) UDR-TD, (**c**,**g**,**k**) CR-RD1, and (**d**,**h**,**l**) CR-RD2.

Figure 11 gives the Schmid factor maps (Figure 11a–h) and statistics of the SFs (Figure 11i–l) for basal <a> slips and prismatic <a> slips in samples with different rolling modes. The critical resolved shear stress (CRSS) of the basal <a> slip was two to four times larger than that of the prismatic <a> slip, and the CRSS of the pyramidal <a> slip was three to six times larger than that of the prismatic <a> slip. The pyramidal <a> slip generally did not occur due to large CRSS during slipping, so only the basal <a> slip and prismatic <a> slip are further discussed [24]. For the UDR-RD and UDR-TD samples in the UDR state, the average SFs for the basal <a> slip were 0.31 and 0.30, respectively, and for the prismatic <a> slip, they were 0.30 and 0.33, respectively. Due to the similar SF values of the basal <a> slip and prismatic <a> slip, the ease of activation of the slip system was the same for the UDR-RD and UDR-TD samples. Therefore, the strength anisotropy of the UDR sample became weaker. For the CR-RD1 and CR-RD2 samples in the CR state, the average Schmidt factors for the basal <a> slip were both 0.37, and for the prismatic <a> slip, they were 0.28 and 0.29, respectively. The SFs were consistent with the UDR sample, leading to similar strength anisotropy in the CR sample.

## 4. Conclusions

In this study, the microstructure characteristics and mechanical properties of TiB-TMC subjected to UDR and CR were comparatively investigated. The present work aimed to investigate the influence of matrix texture and TiB distribution characteristics on the anisotropy of mechanical properties in the TiB-TMC with different rolling methods. The main conclusions can be summarized as follows:

1. The TiB-TMC microstructure in the as-rolled state consisted of α_p_ and β_t_ and both had a weak texture. For the TiB whisker distribution characteristics, the TiB whiskers in the UDR sample were distributed along the rolling direction, and the TiB whiskers in the CR sample were oriented randomly, but the statistical plot of the angle of the TiB whiskers for RD1 showed a weak Gaussian peak.
2. The yield strengths of the UDR-RD and UDR-TD samples were 975 MPa and 970 MPa, respectively, and the elongations were 12.2% and 1.5%, respectively. The UDR sample exhibited significant plastic anisotropy. When the rolling mode changed to CR, the yield strengths of the CR-RD1 and CR-RD2 samples were 997 MPa and 1019 MPa, respectively, and the elongations were 10.6% and 10.2%, respectively. The CR sample had weak strength and plasticity anisotropy.
3. Matrix texture influenced strength anisotropy in the TiB-TMCs. The UDR and CR samples had similar average SF values for the basal <a> slip and prismatic <a> slip in different loading directions due to the lower texture intensity, which led to a similar ease of slip system activation and therefore weaker strength anisotropy.
4. The TiB whisker distribution characteristics determined the plastic anisotropy of the TiB-TMCs. The parallel/perpendicular alignment of the TiB whiskers to the rolling direction led to significant plastic anisotropy in the UDR samples, while the similarly random distribution of the TiB whisker orientation in the CR-RD1 and CR-RD2 samples successfully eliminated such plastic anisotropy. This was mainly because of the synergetic effects of the increased difficulty of matrix crack initiation and the enhanced resistance of matrix crack extension as a result of the lengthened interspacing of the embedded TiB whiskers in both the CR-RD1 and CR-RD2 samples.

## Data Availability

The original contributions presented in this study are included in the article. Further inquiries can be directed to the corresponding author.
